# 2-(1*H*-Imidazo[4,5-*f*][1,10]phenanthrolin-2-yl)phenol monohydrate

**DOI:** 10.1107/S1600536808018527

**Published:** 2008-06-25

**Authors:** Wen-Zhi Zhang, Li Li, Ying-Hui Xiao

**Affiliations:** a College of Chemistry and Chemical Engineering, Qiqihar University, Qiqihar 161006, Heilongjiang Province, People’s Republic of China

## Abstract

The asymmetric unit of the title compound, C_19_H_12_N_4_O·H_2_O, contains one organic molecule and one solvent water mol­ecule, which are connected by N—H⋯O and O—H⋯N hydrogen bonds. In addition, there is one intra­molecular O—H⋯N hydrogen bond. The organic mol­ecule is essentially planar (r.m.s. deviation for all non-H atoms = 0.028 Å).

## Related literature

For related literature, see: Yin (2008 ▶). For a related structure, see: Sun *et al.* (2007 ▶).
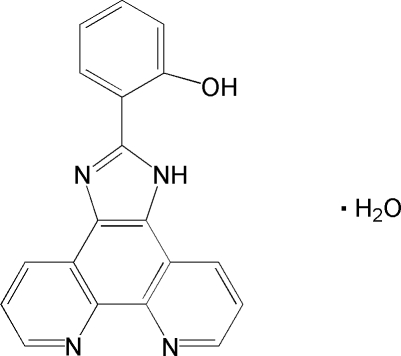


## Experimental

### 

#### Crystal data

C_19_H_12_N_4_O·H_2_O
*M* *_r_* = 330.34
Monoclinic, 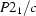

*a* = 4.5272 (9) Å
*b* = 19.822 (4) Å
*c* = 16.956 (3) Å
β = 94.15 (3)°
*V* = 1517.6 (5) Å^3^
*Z* = 4
Mo *K*α radiation
μ = 0.10 mm^−1^
*T* = 293 (2) K
0.21 × 0.17 × 0.15 mm

#### Data collection

Rigaku R-AXIS RAPID diffractometer
Absorption correction: multi-scan (*ABSCOR*; Higashi, 1995 ▶) *T*_min_ = 0.975, *T*_max_ = 0.989
14107 measured reflections
3351 independent reflections
1342 reflections with *I* > 2σ(*I*)
*R*_int_ = 0.177

#### Refinement

*R*[*F* ^2^ > 2σ(*F* ^2^)] = 0.094
*wR*(*F* ^2^) = 0.205
*S* = 1.02
3351 reflections
232 parameters
3 restraints
H atoms treated by a mixture of independent and constrained refinement
Δρ_max_ = 0.20 e Å^−3^
Δρ_min_ = −0.21 e Å^−3^

### 

Data collection: *PROCESS-AUTO* (Rigaku, 1998 ▶); cell refinement: *PROCESS-AUTO*; data reduction: *PROCESS-AUTO*; program(s) used to solve structure: *SHELXS97* (Sheldrick, 2008 ▶); program(s) used to refine structure: *SHELXL97* (Sheldrick, 2008 ▶); molecular graphics: *SHELXTL-Plus* (Sheldrick, 2008 ▶); software used to prepare material for publication: *SHELXL97*.

## Supplementary Material

Crystal structure: contains datablocks global, I. DOI: 10.1107/S1600536808018527/bt2727sup1.cif

Structure factors: contains datablocks I. DOI: 10.1107/S1600536808018527/bt2727Isup2.hkl

Additional supplementary materials:  crystallographic information; 3D view; checkCIF report

## Figures and Tables

**Table 1:** Hydrogen-bond geometry (Å, °)

*D*—H⋯*A*	*D*—H	H⋯*A*	*D*⋯*A*	*D*—H⋯*A*
O1—H1*A*⋯N3	0.82	1.83	2.569 (5)	149
N4—H4⋯O1*W*	0.86	1.90	2.744 (4)	169
O1*W*—H*W*12⋯N2^i^	0.863 (18)	1.91 (2)	2.715 (5)	155 (4)
O1*W*—H*W*12⋯N1^i^	0.863 (18)	2.62 (4)	3.255 (5)	131 (3)

Symmetry code: (i) 
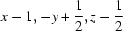
.

**Fig. 1.**
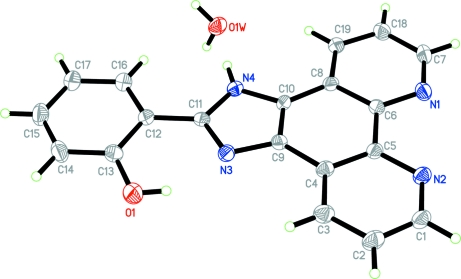
A perspective view of the title compound, showing the atomic numbering scheme. Displacement ellipsoids are drawn at the 30% probability level.

## supplementary crystallographic information

### Comment

1,10-Phenanthroline and its derivatives are commonly used as ligands in metal-organic coordination polymers (Sun *et al.*,  2007; Yin, 2008). The title compound  was synthesized from [4,5-f]1,10-phenanthroline. All  bond lengths are within normal ranges. The H_2_O molecules links the 2-(1H-imidazo[4,5-f][1,10]phenanthrolin-2-yl)phenol molecules by hydrogen bonds to the nitrogen atoms of the imidazo-phenantholine ring systems.

### Experimental

1,10-Phenanthroline-5,6-dione (1.5 mmol) and 2-hydroxybenzaldehyde (1.5 mmol) were dissolved in CH_3_COOHCH_3_COONH_4_ (1:1) solution (30 ml). The mixture was refluxed for 1.5 h under argon, after cooling, this mixture was diluted with water and neutralized with concentrated aqueous ammonia, immediately resulting a yellow precipitate, which was washed with water, acetone and diethyl ether respectively. Crystals of the title compound were obtained by recrystallization from dichloromethane.

### Refinement

C- and N-bound H atoms were positioned geometrically (N-H = 0.86 Å and C-H = 0.93-0.96 Å) and refined as riding, with U_iso_(H) = 1.2U_eq_(carrier). The water H-atoms were located in a difference Fourier map, and were refined with distance restraints of O–H = 0.85±0.01 Å and HW11···HW12 = 1.35±0.01 Å and with U_iso_(H) = 1.2U_eq_(O).

### Crystal data

**Table:**

C_19_H_12_N_4_O·H_2_O	*F*_000_ = 688
*M**_r_* = 330.34	*D*_x_ = 1.446 Mg m^−^^3^
Monoclinic, *P*2_1_/*c*	Mo *K*α radiation λ = 0.71073 Å
Hall symbol: -P 2ybc	Cell parameters from 6908 reflections
*a* = 4.5272 (9) Å	θ = 3.0–27.5º
*b* = 19.822 (4) Å	µ = 0.10 mm^−^^1^
*c* = 16.956 (3) Å	*T* = 293 (2) K
β = 94.15 (3)º	Block, pale yellow
*V* = 1517.6 (5) Å^3^	0.21 × 0.17 × 0.15 mm
*Z* = 4	

### Data collection

**Table:**

Rigaku R-AXIS RAPID diffractometer	3351 independent reflections
Radiation source: rotating anode	1342 reflections with *I* > 2σ(*I*)
Monochromator: graphite	*R*_int_ = 0.177
Detector resolution: 10.0 pixels mm^-1^	θ_max_ = 27.5º
*T* = 293(2) K	θ_min_ = 3.2º
ω scans	*h* = −5→5
Absorption correction: multi-scan(ABSCOR; Higashi, 1995)	*k* = −25→25
*T*_min_ = 0.975, *T*_max_ = 0.989	*l* = −21→21
14107 measured reflections	

### Refinement

**Table:**

Refinement on *F*^2^	Secondary atom site location: difference Fourier map
Least-squares matrix: full	Hydrogen site location: inferred from neighbouring sites
*R*[*F*^2^ > 2σ(*F*^2^)] = 0.094	H atoms treated by a mixture of independent and constrained refinement
*wR*(*F*^2^) = 0.205	*w* = 1/[σ^2^(*F*_o_^2^) + (0.0767*P*)^2^] where *P* = (*F*_o_^2^ + 2*F*_c_^2^)/3
*S* = 1.03	(Δ/σ)_max_ = 0.006
3351 reflections	Δρ_max_ = 0.20 e Å^−^^3^
232 parameters	Δρ_min_ = −0.21 e Å^−^^3^
3 restraints	Extinction correction: none
Primary atom site location: structure-invariant direct methods	

### Special details

**Table:**

Geometry. All esds (except the esd in the dihedral angle between two l.s. planes) are estimated using the full covariance matrix. The cell esds are taken into account individually in the estimation of esds in distances, angles and torsion angles; correlations between esds in cell parameters are only used when they are defined by crystal symmetry. An approximate (isotropic) treatment of cell esds is used for estimating esds involving l.s. planes.
Refinement. Refinement of F^2^ against ALL reflections. The weighted R-factor wR and goodness of fit S are based on F^2^, conventional R-factors R are based on F, with F set to zero for negative F^2^. The threshold expression of F^2^ > 2sigma(F^2^) is used only for calculating R-factors(gt) etc. and is not relevant to the choice of reflections for refinement. R-factors based on F^2^ are statistically about twice as large as those based on F, and R- factors based on ALL data will be even larger.

### Fractional atomic coordinates and isotropic or equivalent isotropic displacement parameters (Å^2^)

**Table:**

	*x*	*y*	*z*	*U*_iso_*/*U*_eq_	
C1	0.8402 (11)	0.1329 (2)	1.0945 (2)	0.0583 (13)	
H1	0.9502	0.1313	1.1429	0.070*	
C2	0.6283 (11)	0.0844 (3)	1.0794 (2)	0.0632 (14)	
H2	0.5933	0.0519	1.1172	0.076*	
C3	0.4704 (10)	0.0846 (2)	1.0080 (2)	0.0568 (12)	
H3	0.3234	0.0526	0.9964	0.068*	
C4	0.5324 (9)	0.1337 (2)	0.9525 (2)	0.0454 (11)	
C5	0.7451 (9)	0.1833 (2)	0.9742 (2)	0.0436 (10)	
C6	0.8102 (9)	0.2370 (2)	0.9201 (2)	0.0434 (10)	
C7	1.0598 (11)	0.3337 (2)	0.8980 (3)	0.0622 (13)	
H7	1.1926	0.3669	0.9163	0.075*	
C8	0.6649 (9)	0.2387 (2)	0.8427 (2)	0.0414 (10)	
C9	0.3919 (9)	0.1377 (2)	0.8750 (2)	0.0419 (10)	
C10	0.4603 (9)	0.1867 (2)	0.8232 (2)	0.0413 (10)	
C11	0.1276 (9)	0.1180 (2)	0.7673 (2)	0.0421 (10)	
C12	−0.0845 (9)	0.0871 (2)	0.7108 (2)	0.0467 (11)	
C13	−0.2259 (10)	0.0279 (2)	0.7296 (3)	0.0544 (12)	
C14	−0.4299 (11)	−0.0019 (3)	0.6760 (3)	0.0685 (14)	
H14	−0.5268	−0.0411	0.6895	0.082*	
C15	−0.4894 (11)	0.0265 (3)	0.6030 (3)	0.0740 (16)	
H15	−0.6268	0.0062	0.5671	0.089*	
C16	−0.1476 (10)	0.1140 (2)	0.6352 (2)	0.0610 (13)	
H16	−0.0506	0.1529	0.6205	0.073*	
C17	−0.3502 (11)	0.0840 (3)	0.5825 (3)	0.0717 (15)	
H17	−0.3924	0.1028	0.5327	0.086*	
C18	0.9353 (10)	0.3391 (2)	0.8210 (2)	0.0587 (13)	
H18	0.9871	0.3745	0.7887	0.070*	
C19	0.7362 (10)	0.2917 (2)	0.7935 (2)	0.0486 (11)	
H19	0.6485	0.2946	0.7423	0.058*	
O1	−0.1730 (7)	−0.00281 (16)	0.80052 (18)	0.0763 (11)	
H1A	−0.0499	0.0189	0.8278	0.114*	
N1	1.0015 (8)	0.28440 (19)	0.94675 (19)	0.0546 (10)	
N2	0.9002 (8)	0.18203 (19)	1.04526 (18)	0.0504 (10)	
N3	0.1823 (7)	0.09440 (17)	0.84045 (18)	0.0449 (9)	
N4	0.2873 (7)	0.17430 (17)	0.75510 (17)	0.0458 (9)	
H4	0.2814	0.1979	0.7125	0.055*	
O1W	0.3484 (8)	0.24505 (18)	0.61814 (17)	0.0688 (10)	
HW12	0.195 (7)	0.257 (2)	0.588 (2)	0.083*	
HW11	0.444 (9)	0.219 (2)	0.590 (2)	0.083*	

### Atomic displacement parameters (Å^2^)

**Table:**

	*U*^11^	*U*^22^	*U*^33^	*U*^12^	*U*^13^	*U*^23^
C1	0.074 (4)	0.061 (3)	0.039 (2)	0.011 (3)	−0.006 (2)	0.001 (2)
C2	0.075 (4)	0.071 (4)	0.044 (3)	0.008 (3)	0.004 (2)	0.013 (2)
C3	0.064 (3)	0.055 (3)	0.052 (3)	0.001 (3)	0.007 (2)	0.008 (2)
C4	0.049 (3)	0.048 (3)	0.039 (2)	0.007 (2)	0.0045 (19)	0.002 (2)
C5	0.046 (3)	0.044 (3)	0.041 (2)	0.008 (2)	−0.0006 (19)	−0.003 (2)
C6	0.045 (3)	0.039 (3)	0.046 (2)	0.005 (2)	0.002 (2)	−0.005 (2)
C7	0.069 (4)	0.053 (3)	0.063 (3)	−0.012 (3)	−0.009 (2)	−0.009 (3)
C8	0.043 (3)	0.040 (3)	0.041 (2)	0.007 (2)	0.0010 (19)	0.0004 (19)
C9	0.040 (3)	0.039 (3)	0.047 (2)	0.001 (2)	0.0010 (19)	0.000 (2)
C10	0.039 (3)	0.042 (3)	0.041 (2)	0.004 (2)	−0.0061 (19)	−0.001 (2)
C11	0.043 (3)	0.035 (2)	0.047 (2)	0.009 (2)	0.0010 (19)	−0.003 (2)
C12	0.044 (3)	0.042 (3)	0.053 (3)	0.004 (2)	−0.004 (2)	−0.002 (2)
C13	0.055 (3)	0.046 (3)	0.062 (3)	0.006 (2)	0.002 (2)	−0.006 (2)
C14	0.060 (4)	0.056 (3)	0.088 (4)	−0.010 (3)	0.001 (3)	−0.022 (3)
C15	0.066 (4)	0.078 (4)	0.075 (4)	−0.003 (3)	−0.016 (3)	−0.022 (3)
C16	0.058 (3)	0.057 (3)	0.066 (3)	−0.001 (3)	−0.009 (2)	−0.003 (3)
C17	0.072 (4)	0.071 (4)	0.069 (3)	−0.001 (3)	−0.019 (3)	−0.007 (3)
C18	0.069 (4)	0.049 (3)	0.057 (3)	−0.007 (2)	0.001 (2)	0.004 (2)
C19	0.054 (3)	0.046 (3)	0.046 (2)	0.001 (2)	0.000 (2)	0.002 (2)
O1	0.077 (2)	0.066 (2)	0.083 (2)	−0.0178 (19)	−0.0115 (18)	0.0141 (19)
N1	0.063 (3)	0.047 (2)	0.053 (2)	−0.011 (2)	−0.0019 (18)	−0.0013 (19)
N2	0.059 (2)	0.052 (2)	0.0394 (19)	0.0082 (19)	0.0000 (17)	0.0002 (18)
N3	0.043 (2)	0.046 (2)	0.0449 (19)	0.0014 (18)	0.0012 (16)	0.0004 (17)
N4	0.046 (2)	0.044 (2)	0.0463 (19)	0.0008 (18)	−0.0033 (16)	0.0060 (17)
O1W	0.077 (3)	0.071 (3)	0.0559 (19)	0.000 (2)	−0.0092 (16)	0.0158 (17)

### Geometric parameters (Å, °)

**Table:**

C1—N2	1.323 (5)	C11—N3	1.332 (5)
C1—C2	1.370 (6)	C11—N4	1.353 (5)
C1—H1	0.9300	C11—C12	1.443 (5)
C2—C3	1.360 (5)	C12—C13	1.384 (6)
C2—H2	0.9300	C12—C16	1.400 (5)
C3—C4	1.397 (5)	C13—O1	1.353 (5)
C3—H3	0.9300	C13—C14	1.380 (6)
C4—C5	1.407 (6)	C14—C15	1.368 (6)
C4—C9	1.420 (5)	C14—H14	0.9300
C5—N2	1.351 (4)	C15—C17	1.360 (7)
C5—C6	1.449 (5)	C15—H15	0.9300
C6—N1	1.334 (5)	C16—C17	1.370 (6)
C6—C8	1.424 (5)	C16—H16	0.9300
C7—N1	1.318 (5)	C17—H17	0.9300
C7—C18	1.389 (5)	C18—C19	1.361 (6)
C7—H7	0.9300	C18—H18	0.9300
C8—C19	1.393 (5)	C19—H19	0.9300
C8—C10	1.410 (5)	O1—H1A	0.8200
C9—C10	1.360 (5)	N4—H4	0.8600
C9—N3	1.378 (5)	O1W—HW12	0.863 (18)
C10—N4	1.370 (4)	O1W—HW11	0.841 (18)
			
N2—C1—C2	124.9 (4)	C13—C12—C16	117.8 (4)
N2—C1—H1	117.5	C13—C12—C11	120.3 (4)
C2—C1—H1	117.5	C16—C12—C11	121.8 (4)
C3—C2—C1	118.7 (4)	O1—C13—C14	117.4 (4)
C3—C2—H2	120.7	O1—C13—C12	122.0 (4)
C1—C2—H2	120.7	C14—C13—C12	120.6 (4)
C2—C3—C4	118.9 (4)	C15—C14—C13	119.9 (5)
C2—C3—H3	120.5	C15—C14—H14	120.1
C4—C3—H3	120.5	C13—C14—H14	120.1
C3—C4—C5	118.6 (4)	C17—C15—C14	120.8 (5)
C3—C4—C9	124.4 (4)	C17—C15—H15	119.6
C5—C4—C9	117.1 (4)	C14—C15—H15	119.6
N2—C5—C4	121.4 (4)	C17—C16—C12	121.0 (5)
N2—C5—C6	117.6 (4)	C17—C16—H16	119.5
C4—C5—C6	121.0 (3)	C12—C16—H16	119.5
N1—C6—C8	122.8 (4)	C15—C17—C16	119.8 (5)
N1—C6—C5	117.3 (3)	C15—C17—H17	120.1
C8—C6—C5	119.9 (4)	C16—C17—H17	120.1
N1—C7—C18	124.1 (4)	C19—C18—C7	118.8 (4)
N1—C7—H7	117.9	C19—C18—H18	120.6
C18—C7—H7	117.9	C7—C18—H18	120.6
C19—C8—C10	126.0 (3)	C18—C19—C8	119.5 (4)
C19—C8—C6	117.2 (4)	C18—C19—H19	120.3
C10—C8—C6	116.8 (4)	C8—C19—H19	120.3
C10—C9—N3	110.6 (3)	C13—O1—H1A	109.5
C10—C9—C4	122.0 (4)	C7—N1—C6	117.6 (4)
N3—C9—C4	127.4 (4)	C1—N2—C5	117.4 (4)
C9—C10—N4	105.8 (3)	C11—N3—C9	104.6 (3)
C9—C10—C8	123.2 (3)	C11—N4—C10	107.3 (3)
N4—C10—C8	130.9 (4)	C11—N4—H4	126.3
N3—C11—N4	111.5 (3)	C10—N4—H4	126.3
N3—C11—C12	122.5 (4)	HW12—O1W—HW11	105 (3)
N4—C11—C12	125.9 (4)		

### Hydrogen-bond geometry (Å, °)

**Table:**

*D*—H···*A*	*D*—H	H···*A*	*D*···*A*	*D*—H···*A*
O1—H1A···N3	0.82	1.83	2.569 (5)	149
N4—H4···O1W	0.86	1.90	2.744 (4)	169
O1W—HW12···N2^i^	0.863 (18)	1.91 (2)	2.715 (5)	155 (4)
O1W—HW12···N1^i^	0.863 (18)	2.62 (4)	3.255 (5)	131 (3)

Symmetry codes: (i) *x*−1, −*y*+1/2, *z*−1/2.
